# Effectiveness of stellate ganglion block for the treatment of patulous eustachian tube: A case report

**DOI:** 10.1002/ccr3.6713

**Published:** 2023-01-03

**Authors:** Junko Akiyama, Mina Imai, Keisuke Yamaguchi

**Affiliations:** ^1^ Department of Anesthesiology and Pain Medicine Juntendo Tokyo Koto Geriatric Medical Center Koto‐Ku Japan; ^2^ Department of Anesthesiology Saiseikai Kawaguchi General Hospital Kawaguchi Japan

**Keywords:** autophony, breathing awareness, patulous eustachian tube, stellate ganglion block

## Abstract

A 56‐year‐old woman presented with a 2 months history of patulous eustachian tube. She had sudden weight loss after developing a cold, after which she had been experiencing disabling autophony and a sensation of blockage in the ear. She underwent stellate ganglion block in 8 months; her symptoms resolved subsequently.

## Abstract 2

We concluded that successful management of PET can be obtained with SGB; this treatment was not previously used for this disorder.

## 1 INTRODUCTION

The eustachian tube (ET) allows pressure equalization between the mesotympanum and the nasopharynx and maintains the health of the middle ear cavity. A patulous eustachian tube (PET) is abnormally patent, with a reported incidence of 0.3%–6.6%.^1^ PET symptoms occur due to the ET remaining open for an extended time; they include enhanced awareness of one's own voice and breathing, a phenomenon known as autophony, and a continuous sensation of ear blockage. In severe cases, these symptoms can lead to depression and suicidal tendencies.^2^ Thus, PET is a benign disease that can nevertheless seriously affect the quality of life.

The ET is typically closed at rest and maintains middle ear ventilation to facilitate sound transmission from the tympanic membrane to the cochlea. Various risk factors are associated with PET, including rapid weight loss (the most common cause), pregnancy, aging, and hypotension. Anatomical causes of PET include the loss of subcutaneous peritubal tissue (Ostmann's fat pad), abnormal contractile activity of the peritubal muscles (soft palate tensor and levator muscles and salpingopharyngeus muscle), or an inability of the pterygoid venous plexus to effectively close the ET.^3^

At present, no treatment for PET has been established. Common therapeutic measures include lifestyle modifications, such as avoiding rapid weight loss, coffee/caffeine, stress, and anxiety; noninvasive treatments (nasal instillation of physiological saline^4^ and infusion of an absorbable gelatin sponge solution into the pharyngeal ET orifice^5^); and medical management with adenosine triphosphate (ATP) infusion and oriental medicine.^6^ There are numerous reports of treatment with oriental medicine in Japan, and Kamikihito, which has peripheral vasodilatory and anti‐stress effects, is often used for PET.^7^ ATP increases the auditory tube closing pressure by stimulating the vascular flow. In addition, several surgical or interventional treatment options are available for patients with persistent symptoms. Surgical options include the insertion of a tympanostomy tube, heavy tympanic membrane loading, a tympanic passage plug, or nasal injection of a soft‐tissue bulking agent into the tubular torus.^8^ The mechanism underlying these surgical options involves the physical narrowing of the auditory tube opening. Thus, these treatments improve PET symptoms by facilitating ET closure.

The stellate ganglion block (SGB) has been used to treat several clinical sympathetic pain and vascular insufficiency syndromes. The stellate ganglion is formed by the fusion of the inferior cervical and superior thoracic sympathetic ganglions. SGB improves the blood supply to the head and neck. Therefore, we believe that SGB can also alleviate PET symptoms by increasing the blood supply.

Here, we report a successful case of PET treatment using SGB. To the best of our knowledge, this treatment has not been previously attempted in PET cases.

## 2 CASE REPORT

A 56‐year‐old woman diagnosed with PET visited our hospital. She was 158 cm tall and weighed 55 kg. She had previously attended our clinic for back pain. Two months prior to the visit, she had caught a cold and experienced rapid weight loss. Since then, she had been experiencing disabling autophony and a sensation of ear blockage. These symptoms were reduced in the supine and lordotic positions. Soon after, she was diagnosed with PET through otoscopy by an otolaryngologist and was treated with the oriental medicine “Kamikihito,” a popular Japanese herbal medicine used for PET. However, her symptoms continued to persist, and she consulted our hospital, where she had been previously treated for lumbago for 2 years.

**FIGURE 1:**
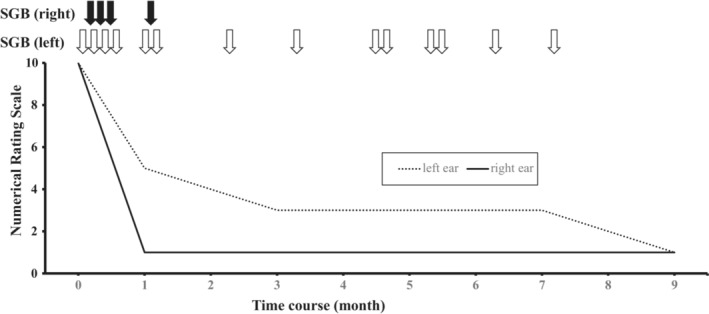
Time course of treatment and improvement of pain according to the numerical rating scale. We performed SGB for a total of 4 times on the right side and 14 times on the left side of the patient. SGB: stellate ganglion block.

Her symptoms were severe, reaching a score of 10 on the numerical rating scale (NRS), which ranges from 0 to 10, with 0 representing “no autophony” and 10 being “the worst autophony imaginable” according to her subjective judgment. We offered SGB as treatment and, after explaining the procedure, obtained informed consent from the patient. As with most SGBs performed at our hospital, the procedure was performed under ultrasound‐guidance for safety reasons. We used a micro‐convex probe to identify the longus colli muscle at C6 and inject it with the local anesthesia. Ultrasound‐guided SGB was performed by injecting 3 ml of 1% mepivacaine. We chose this drug for SGB because mepivacaine is relatively safe and commonly used at our outpatient pain clinic. The treatment was performed on alternate sides in each session, and the NRS score for symptoms in the right ear decreased to 1 after the first procedure. We continued to perform SGB twice a week for a month, and the NRS score for the left side decreased to 3. Subsequently, the frequency of SGB was reduced to once per month. After 8 months, the NRS score for the left side had decreased to 1, and we completed the treatment after 18 sessions because the patient's complaint improved, and she was satisfied with her condition. Complications like inadvertent epidural injection, subarachnoid hemorrhage, accidental intravascular injection, hematoma formation, and esophageal injury were not observed. The procedure was completed safely without any severe complications (Figure 1).

## 3 DISCUSSION

This report describes the first successful treatment of PET with SGB, an intervention typically used to treat various pain conditions in the head and neck, such as postherpetic neuralgia and complex regional pain syndrome. In particular, SGB is used to treat vascular insufficiency. Several studies have reported that SGB reduces vascular resistance, increases blood flow in the homolateral common carotid and vertebral arteries, and decreases the contralateral blood flow.^9^, ^10^ Ultrasound‐guided SGB has been reported to allow an effective and precise sympathetic block while requiring only a low injectate volume, thereby minimizing the risk of vascular and soft‐tissue injuries.^11^ PET symptoms often diminish in the supine and lordotic positions; this effect is mainly related to the enlargement of the pterygoid venous plexus (PVP).^12^ These positions cause venous pooling in the PVP, swelling the tensor veli palatini and medial pterygoid muscles. PET treatments aim to increase blood flow in the areas surrounding the ET, hence increasing the volume of the nasopharyngeal ET. SGB has similar effects on blood flow in the head and neck. In this case, the PVP blood flow was likely increased by the SGB, reducing the auditory tube lumen. This mechanism may have reduced the patient's PET symptoms.

This case report had some limitations; the main one was the lack of an auditory tube function test. PET diagnostic criteria have not yet been definitively established; however, the Japan Otological Society's Eustachian Tube Committee proposed a few criteria in 2016 (Table 1).^13^ These criteria include (1) subjective symptoms, (2) improvement by tubal obstruction procedures, and (3) objective findings. If all three criteria are met, PET can be a definite diagnosis; if only two criteria are met (1 and either 2 or 3), PET is a possible diagnosis. In this case, all three criteria were present: The patient presented with voice autophony and aural fullness; the symptoms were reduced in the supine or lordotic position, and respiratory fluctuation of the tympanic membrane was noted. However, an evaluation with tubo‐tympano‐aerodynamic‐graphy and sonotubometry is essential for the assessment of the treatment progress. Although our evaluations were sufficient to diagnose PET, an auditory tube function test would have allowed an objective PET evaluation to determine its response to treatment and follow‐up.

**TABLE 1:** Diagnostic criteria for patulous eustachian tube proposed by the Japan Otological Society

1. Subjective symptoms
One or more of the following symptoms: voice autophony, a sense of aural fullness, and breathing autophony
2. Tubal obstruction procedures (A or B) clearly improve the symptoms
A. Posture change in the lying/lordotic position
B. Pharyngeal orifice obstruction treatment (swab, gel, etc.)
3. At least one of the following objective findings for a patent E‐tube:
A. Respiratory fluctuation of the tympanic membrane
B. Variations of external auditory meatus pressure synchronized with the nasopharyngeal pressure
C. Sonotubometry showing (1) test tone sound pressure level less than 100 dB or (2) an open plateau pattern.

*Note*: If all three criteria are met, the diagnosis is “Definite PET”; if only two criteria are met (1 and either 2 or 3), the diagnosis is “Possible PET.” PET: Patulous Eustachian tube.

Treatment options for PET are diverse, including minimally invasive therapy, medical management, and surgery. The minimally invasive treatment options include increasing fluid intake, use of nasal distilled water, and putting one's head between one's legs. The success rate of pharmacologic treatment is 60%–100%, although the underlying research evidence is very poor. Surgical treatments can target the middle ear, and the most commonly used middle ear intervention is the insertion of a ventilation tube.^14^ In this case, SGB was chosen because it is a minimally invasive therapy, pharmacological therapy was not effective, and the patient was not willing to undergo surgery.

In conclusion, this report describes the first successful management of PET with SGB. This procedure may be an effective treatment for PET, and ultrasound‐guided SGB is safer than the conventional technique. However, additional research is required to verify the validity of this technique. The SGB may deserve to be studied as a therapeutic option for PET in properly designed studies, as the disease has no established treatment, and SGB was feasible in this case.

## AUTHOR CONTRIBUTIONS

KY was responsible for obtaining consent, discussions with the ethics committee, acquiring the data, and drafting the manuscript. MI and JA established the clinical diagnosis with KY and assisted in the drafting and critical revision of the manuscript. MI critically revised the manuscript. All the authors approved the final version of the manuscript.

## CONFLICT OF INTEREST

The authors certify that there is no conflict of interest with any financial organization regarding the material discussed in the manuscript. The patient has consented to the submission of the case report to the journal.

## ETHICAL APPROVAL

As a single case report with the patient's signed consent, no other ethical review was required.

## CONSENT

Written informed consent was obtained from the patient for publication of this case report.

## Data Availability

Data are available on reasonable request.
